# Supplementary material for: Therapeutic Effects of the Pilates Method in Patients with Multiple Sclerosis: A Systematic Review
Source: J Clin Med. 2022 Jan 28;11(3):683. doi: 10.3390/jcm11030683 (PMC8836864; doi:10.3390/jcm11030683)
Supplement: Supplementary file 1 [file jcm-11-00683-s001.zip › jcm-1515151-supplementary.pdf]

Table S1. Characteristics of the reviewed studies.

Study Year	Purpose	Subjects Mean age±sd (range)	Type of Intervention (EG/CG)	Dropouts during intervention	Adherence/ Attendance (range)	Study variables (measurement tools)	Assessments (weeks)	Findings	Adverse effects
Güngör et al. [28] 2021	To investigate the effects of Pilates-based core stability training given as supervised or home-based on lower extremity strength and postural control in pwMS	EG1=(25 initially) 22 (20♀2♂) 41.2y±9.9 (20-57)<br><br>EG2=(25 initially) 20 (16♀4♂) 37.5y±11.9 (22-58)	EG1: supervised Pilates training<br><br>EG2: home-based Pilates training	3 in EG1 (2 transportation problems, 1 Covid-19) + 5 in EG2 (1 relapse, 3 not doing regularly, 1 Covid-19)	NA	Isokinetic knee muscle strength (isokinetic dynamometer), postural sway (stabilometric platform), core endurance (trunk flexor/extensor/lateral endurance tests) and strength (curl-up and modified push-up tests), physical capacity (2MWT, TUG), fatigue (FSS)	0, 8	• EG1 and EG2: ↑ in all measures (except some sub-parameters of postural sway in EG2).<br>• EG1 better than EG2: ↑ in all measures (except in postural sway in the closed eyes-foam surface and the medial-lateral direction, and in fatigue)	1 relapse (EG2)
Fleming et al. [29] 2021	To quantify the effects of 8-weeks of home-based Pilates compared to wait-list control on anxiety, depressive and fatigue symptoms among pwMS	EG=(39 initially) 29 (27♀2♂) 45.3y±8.6<br><br>CG=(41 initially) 34 (27♀7♂) 48.2y±9.76	EG: home-based Pilates guided by a DVD<br><br>CG= wait-list	10 in EG (3 no reply, 3 unable to commit at that time, 2 exercise level was difficult, 2 personal reasons unrelated to the study) + 7 in CG (6 no reply, 1 personal reasons)	NA	Fatigue (MFIS), Anxiety (STAY-Y2, HADS-A), depression (QIDS, HADS-D), physical activity (7d-PAR, GLTEQ)	0, 2, 4, 6, 8	• 7d-PAR and weekly GLTEQ were significantly higher in the CG at baseline<br>• EG: ↓ depression symptoms, anxiety and fatigue (physical, cognitive, psychosocial and total); symptoms improvements ranged from moderate-to-large effects; clinically meaningful improvements in depressive and fatigue symptoms<br>• EG better than CG: depression symptoms, anxiety and fatigue	No
Gheitasi et al. [30] 2021	To evaluate the effect of Pilates exercises on the functional balance of male pwMS	EG=15 (15♂)<br>CG=15 (15♂)<br><br>31.35y±5.7 (25-40)	EG: Pilates<br><br>CG: usual medical care	No	NA	Balance (BBS, TUG, FRT)	0, 12	• EG: ↑BBS, TUG and FRT<br>• EG better than CG: ↑ in all measures	No
Arntzen et al. [31] 2020	To investigate the immediate and long-term effects of a Pilates intervention compared with those of standard care on walking	EG=39 (27♀12♂) 52.2y±12.9 (24-77)<br><br>CG=40 (29♀11♂) 48y±8.75 (31-67)	EG: Pilates<br><br>CG: standard care	5 in CG (1 illness, 4 missed) at the 18-week assessment; 2 in EG (missed) + 2 in CG (missed) at the 30-week assessment	2.50±0.16 sessions/week ≈ 83.33%	Walking distance (2MWT), Walking speed (10MWT), Perceived limitations while walking (MSWS-12), Perceived Changes in walking (PGIC-walking), Individual's gait quality (RVGA) and accelerometer monitoring of community ambulation and physical activity (ActiGraph).	0, 7, 18, 30	• EG better than CG: ↑ 2MWT and PGIC at all follow-up time points, 10MWT and MSWS-12 at 7 and 18 weeks, and RVGA at 7 weeks	1 sensorial exacerbation in EG
Ozkul et al. [32] 2020	To investigate the effect of combined exercise training on different cognitive functions in pwMS with cognitive impairment	EG=17 (13♀4♂) 35.88y±9.74<br><br>CG=17 (13♀4♂) 36.76y±9.02	EG: aerobic training + Pilates training<br><br>CG= home relaxation exercises	No	85.29±13.19% (62.5-100, an average of 85.29±13.19%)	Cognitive functions (BRB-N tests), walking capacity (6MWT), fatigue (FIS), mood (BDI), quality of life (MSQoL-54)	0, 8	• EG: ↑verbal memory, visuospatial memory, verbal fluency, information processing speed, walking capacity, fatigue, and quality of life.<br>• CG: ↑verbal memory<br>• EG better than CG: ↑ verbal memory, walking capacity, cognitive fatigue, and physical quality of life<br>• Change in visuospatial memory was positively and weakly associated with the change in mental QoL; Change in verbal fluency and processing speed were negatively and weakly associated with the change in mood	No

Study Year	Purpose	Subjects Mean age±sd (range)	Type of Intervention (EG/CG)	Dropouts during intervention	Adherence/ Attendance (range)	Study variables (measurement tools)	Assessments (weeks)	Findings	Adverse effects
Banitalebi et al. [33] 2020	To investigate the effect of a 12-week (3sessions/week) supervised multimodal exercise program on neurotrophic factors levels	EG=47 (47♀) (18-50y)<br><br>CG=47 (47♀) (18-50y)	EG: exercise program (resistance, endurance, Pilates, balance and stretch exercises)<br><br>CG= home relaxation exercises	1 in EG (due to unwilling) + 4 in CG (3 due to unwilling and 1 to relapse)	NA	Resting level of neurotrophic factors - serum level of BDNF, NT-3, NT4/5, GDNF and CNTF- (blood sample), aerobic capacity (peak oxygen uptake on treadmill), strength (leg extension 1RM), and energy cost of walking (PCI)	0, 12	• EG better than CG: ↑ BDNF, NT-3 and NT-4/5 levels, aerobic capacity and 1RM with exercise<br>• EG with low and disability had significant larger changes in NT-3 compared with CG; significant positive correlation between changes peak oxygen uptake and changes BDNF and NT-4/5 with exercise; 1RM changes with exercise had a significant positive association with BDNF and NT-3	NA (It's no clear)
Abasiyanik et al. [34] 2020	To investigate the effects of Clinical Pilates training on balance, walking, fall risk, respiratory, and cognitive functions in pwMS compared with an active comparator (home exercise training)	EG= (21 initially) 16 (12♀4♂) 42.50y±6.76<br><br>CG= (21 initially) 17 (11♀6♂) 48.24y±11.79	EG: Pilates exercises<br><br>CG= home exercises	5 in EG + 4 in CG	93% in EG, 82% in CG	walking endurance (6MWT), gait speed (T25FW), mobility (TUG), walking impairment (MSWS-12), core stability (curl-up test), balance performance (balance platform), level of perceived balance confidence (ABC), falls risk (FES-I), abdominal muscle endurance (curl-up test) respiratory muscle strength (maximum inspiratory and expiratory pressure using a manovacuometer), and cognitive functions (BICAMS)	0, 8	• EG and CG (postintervention): ↑ walking endurance, gait speed, mobility, walking impairment, core stability, abdominal muscle endurance, and expiratory muscle strength<br>• Only EG (postintervention): ↑ postural stability, inspiratory muscle strength, and cognitive functions, and ↓ fall risk<br>• EG better than CG: ↑ walking endurance, postural stability, core stability, respiratory muscle strength, and cognitive functions	1 fall (CG)
Fleming et al. [35] 2019	To explore the feasibility of an 8-week supervised or home-based Pilates intervention compared to wait-list control on mental health outcomes among pwMS	EG1=5 (5♀) 53.8y±7.95<br><br>EG2=6 (5♀) 46.0y±9.4<br><br>CG=7 (6♀,1♂) 51.3y±6.8	EG1: supervised Pilates<br><br>EG2: home-based Pilates guided by a DVD<br><br>CG= wait-list	2 in EG1 (workplace injury, medication issues) + 1 in CG (one male was omitted from data analyses)	>80% in EG1 and EG2; 100% in CG	Fatigue (MFIS), mood (POMS-B), Anxiety (STAY-Y1, HADS), depression (HADS, QIDS), hours of sleep and activity (7d-PAR), physical activity (GLTEQ), feasibility, retention, attendance, and adverse events	0, 2, 4, 6, 8	• Pilates compliance was high across groups (>80%)<br>• EG1 and EG2 better than CG: ↓ feelings of tension<br>• EG2 better than CG: ↓ feelings of depressed mood al weeks 4, 6 and 8, physical symptoms of fatigue at weeks 4 and 8, and total fatigue at weeks 4 and 8<br>• EG1 worse than EG2: ↑anxiety symptoms	No
Eftekhari and Etemadifar [36] 2018	To investigate the effect of clinical mat Pilates on anthropometric variables, functional indices (balance, walking speed, endurance), and fatigue in females suffering from MS	EG=(15 initially) 13 (13♀) 34.46y±7.29<br><br>CG=(15 initially) 12 (12♀) 31.41y±8.89	EG: Pilates training<br><br>CG: continued their lifestyle	2 in EG + 3 in CG	NA	Height (wall-mounted ruler), body weight (portable scale), body circumferences (measuring tape), skinfold (caliper), fat percentage and body density (Jackson/Pollock 7-site skinfold equation), balance (BBS), gait speed (10MWT), walking endurance (6MWT), fatigue (MFIS).	0, 8	• EG: ↓ in body weight, in fat mass, in waist, hip and mid-arm muscle circumferences, in chest, abdominal, triceps and suprailiac skinfold, in body mass index, in fat percentage, and fatigue, and ↑ body density, balance, gait speed, and walking endurance<br>• EG better than CG: ↑ in all of this variables except triceps skinfold	NA
Ozkul tel al. [37] 2018	To investigate the effect of an 8-week combined exercise training consisting of aerobic and Pilates training on serum concentrations of SOCS1, SOCS3, and BDNF in pwMS and determine the effects of the combined exercise training on physical performance such	EG= (initially 21) 18 (14♀4♂) 34.5y (26-43.25)<br><br>CG= (initially 20) 18 (14♀4♂) 34y (32-43.75)<br><br>HC=18 (14♀4♂) 33y (26.75-43.25)	EG: aerobic training + Pilates training<br><br>CG= home relaxation exercises<br><br>HC: healthy control (to compare their	3 in EG (1 suffered a relapse + 2 removed for work intensity) + 2 in CG (they could not come back to the last measurement)	66-100% in EG (an average of 85%)	Serum concentrations of SOCS1, SOCS3, and BDNF (blood sample), balance (balance platform), functional exercise capacity (6MWT), fatigue (FSS)	0, 8	• EG: ↑ serum BDNF level, balance, and functional exercise capacity, and ↓ fatigue severity<br>• CG: ↑ serum SOCS1 level, and fatigue severity	1 suffered a relapse + 2 removed for work intensity

Study Year	Purpose	Subjects Mean age±sd (range)	Type of Intervention (EG/CG)	Dropouts during intervention	Adherence/ Attendance (range)	Study variables (measurement tools)	Assessments (weeks)	Findings	Adverse effects
	as balance, functional exercise capacity and fatigue		values of biomarkers groups with pwMS)						
Duff et al. [38] 2018	To determine the effect of Pilates on walking performance in pwMS	EG=15 (12♀3♂); CG=15(11♀4♂) 45.4y±8.3	EG: Pilates + a weekly 1-hour massage therapy CG: a weekly 1-hour massage therapy	No (but 1 in EG and 2 in CG not complete postintervention testing due to medical reasons, but they were included in the final analyses)	84.8%±15.5 in EG	Walking ability (6MWT), Functional ability (TUG), Balance (Fullerton Advanced Balance Scale), Flexibility (SRT), Body composition (dual-energy X-ray absorptiometry), Core endurance (plank-hold test), Quadriceps strength (maximum voluntary contraction), Fatigability (sustained maximum voluntary contraction torque drop), Voluntary muscle activation (interpolated twitch technique), daily/weekly physical activity (accelerometers), QoL (MSQoL-54)	0,12	• EG better than CG: ↑Walking and functional ability (TUG with a left turn)<br>• No difference between groups for any other measure	No
Eftekhari and Etemadifar [39] 2018	To investigate the effect of Mat Pilates on the IL-10 and BDNF levels in pwMS	EG=(15 initially) 13 (13♀) 34.46y±7.29 CG=(15 initially) 12 (12♀) 31.41y±8.89	EG: Pilates training CG: continued their lifestyle	2 in EG + 3 in CG	NA	Height (wall-mounted ruler), body weight (portable scale), serum levels of IL-10 and BDNF (blood sample).	0, 8	• EG better than CG: ↑ BDNF	NA
Kalron et al. [40] 2017	To examine the effects of a 12-week Pilates exercise training program on gait and balance in pwMS and compare these results to those of a standard physical therapy intervention program	GE=(25 initially) 22 CG=(25 initially) 23 (final sample 45, 29♀16♂) 43.3y±11.6	EG1: Pilates + 15-minute daily home exercise program CG: standardized program of exercise therapy + 15-minute daily home exercise program	3 in EG + 2 in CG (in all cases due to difficulties in arriving to the MS Center)	NA	Clinical gait and balance tests (TUG, 2MWT, 6MWT, FRT, BBS, and FSST), perceived impact of MS on walking ability (MSWS-12), Fatigue (MFIS), laboratory balance and gait spatio-temporal variables (computer controlled treadmill), and postural control (a sequence of 3 consecutive tests under 2 conditions: OE and CE).	0,12	• EG and CG (postintervention): ↑ 2MWT, 6MWT, TUG, FRT, FSST and MSWS-12, mean step length, and mean single support phase, and ↓ mean step time, time when both legs were in contact with the floor, and center of pressure and sway rate with OE<br>• No difference between groups	No
Bulguroglu et al. [41] 2017	To analyze and compare the effects of Mat Pilates and Reformer Pilates methods on balance, core stability, mobility, fatigue and quality of life in pwMS	EG1=12 45y (39.3-49.5) EG2=13 37y (29.5-40) CG=13 40y (26-43) (all age data are given as median)	EG1: Mat Pilates EG2: Reformer Pilates CG: Breathing and relaxation home exercises	7 (due not able to continue the exercises)	NA	Balance (SLS and ABC), Functional mobility (TUG), Core stability (with several tests: Core endurance measures, Side bridge test, Modified Biering-Sorensen test, Trunk flexion test, Prone bridge test, Core power measures, Sit-ups test, Modified push-ups test), Fatigue severity (FSS), and QoL (MSQoL-54)	0, 8	• EG1 and EG2 (postintervention): ↑ SLS, ABC, TUG, core stability test scores, and mental and physical health dimensions of MSQoL-54, and ↓ FSS<br>• CG (postintervention): ↑ physical health dimension of MSQoL-54<br>• No difference between EG, except in Trunk flexion test (EG2 better than EG1)	NA

Study Year	Purpose	Subjects Mean age±sd (range)	Type of Intervention (EG/CG)	Dropouts during intervention	Adherence/ Attendance (range)	Study variables (measurement tools)	Assessments (weeks)	Findings	Adverse effects
Kara et al. [42] 2017	To compare the effects of aerobic exercise and Pilates exercises on disability, cognition, physical performance, balance, depression and fatigue in pwMS as compared to healthy controls	EG1= (28 initially) 26 (17♀9♂) 43.03y±10.26;<br><br>EG2=(27 initially) 9 (6♀3♂) 49.77y±8.95;<br><br>CG=21 (13♀8♂) 44.42y±5.98	EG1: aerobic exercises;<br><br>EG2: Pilates exercises;<br><br>CG: healthy controls	2 EG1 (exacerbation of symptoms) + 18 EG2 (5 losses to follow-up + 4 exacerbation of symptoms + 7 not coming to second assessment + 2 not coming to exercises programme)	NA	disability (TUG), cognition (MSFC, scale with 3 sub-parameters: T25FW, 9HPT and PASAT-3 version), physical performance (Time roll from right to left, Lie/sit, Sit and stand, and Repeated sitting), balance (BBS), depression (BDI) and fatigue (FIS)	0, 8	• EG1 (postintervention): ↑ all parameters except MSFC-T25FW, BBS and BDI<br>• EG2 (postintervention): ↑ all parameters except MSFC-9HPT and T25FW, PASAT, BBS, BDI and FIS<br>• Postintervention, no difference between EG1 and CG in BDI and FIS (yes in the remaining parameters)<br>• Postintervention, no difference between EG2 and CG in BDI (yes in the remaining parameters)<br>• EG2 better than EG1 (postintervention) in 9HPT with non-dominant hand and PASAT<br>• Positive correlation between EDSS and 9HPT in EG1, and EDSS and PASAT in EG2	Exacerbatio n of symptoms (2 in EG1 + 4 in EG2)
Fox et al. [43] 2016	To compare the effectiveness of a 12-week program of Pilates with relaxation exercise + to compare a 12-week program of standardized exercises with relaxation and to compare Pilates with standardized exercises	EG1=33 (28♀5♂) 53.97y±9.19 (31-73)<br><br>EG2=(35 initially, 25♀10♂) 32 54.60y±11.54 (35-77)<br><br>CG=(initially 32, 21♀11♂) 29 54.13y±10.14 (31-77)	EG1: Pilates exercises<br><br>EG2: Standardized exercises<br><br>CG: relaxation (+15min home exercises in all groups)	3 EG2 (1 fractured humerus + 1 no want to travel to center + 1 NA) + 3 GC (2 adverse events + 1 wife unwell) (all cases, unrelated to study)	66% (EG1), 84% (EG2) and 92% (CG). Adherence to home exercises: 80% (EG1), 78% (EG2) and 91% (CG).	Walking speed (10MWT), balance impairment (FRT-forward and lateral-), walking impairment (MSWS-12), perceived balance confidence (AFC), measure of common dual-task problem (NRS), adherence data (diary)	0,12, 16	• At 12 and 16 weeks, no difference between EG1 and CG for any study variables<br>• EG2 better than CG at 12 weeks in walking speed, forward functional reach, and MSWS-12 and ABC scores<br>• EG2 better than CG at 16 weeks in walking speed, lateral functional reach, and MSWS-12 score<br>• EG2 better than EG1 at 12 weeks for walking speed and MSWS-12 score, and only for MSWS-12 at 16 weeks	No
Küçük et al. [44] 2016	To analyze the effects of clinical Pilates on body control, balance, quality of life, fatigue, and cognition in MS patients	(Initially, 37 patients; finally, 20, 13♀7♂)<br><br>EG=11 53.97y±9.19 (31-73)<br><br>CG=9 54.13y±10.14 (31-77)	EG: Pilates;<br><br>CG: traditional exercises	17 (due to health, transportation, or other problems)	NA	Cognitive impairments (MSFC, scale with 3 sub-parameters: T25FW, 9HPT and PASAT), static and dynamic balance (BBS), physical performance (Time to roll from right to left, Lie/sit test, Sit/stand test, Repeated sit/stand, and 50-foot walking test), walking performance (TUG), static and dynamic sitting balance and trunk coordination and control in a sitting position (TIS), fatigue (MFIS), QoL (MusiQol), and depression (BDI)	0, 8	• EG (postintervention): ↑ balance, physical performance, fatigue, and PASAT step of the MFCS<br>• CG (postintervention): ↑ physical performance (except time to left), walking performance and 9HPT step of the MSFC<br>• EG better than CG: PASAT step of the MSFC and QoL	NA
Hosseini Sisi et al. [45] 2014	To assess effect of 8 weeks of rebound therapy and Pilates practices on static and dynamic balances in males with MS	(all men)<br><br>EG1=15 32.21y±7.6 (22-48)<br><br>EG2=15 30.32y±8.32 (25-50)	EG1: rebound therapy<br><br>EG2: Pilates<br><br>CG: -	NA	NA	Static balance (BBS), dynamic balance (TUG)	0, 8	• EG1 and EG2 (postintervention): ↑ static and dynamic balance<br>• No difference between EG1 and EG2	NA

Study Year	Purpose	Subjects Mean age±sd (range)	Type of Intervention (EG/CG)	Dropouts during intervention	Adherence/ Attendance (range)	Study variables (measurement tools)	Assessments (weeks)	Findings	Adverse effects
		CG=15<br>31.43y±7.09 (24-46)							
Guclu-Cunduz et al. [46]<br>2014	To investigate the effects of Pilates on balance, mobility, and strength in ambulatory pwMS	(all women)<br><br>EG=18<br>36y (29-40)<br><br>CG=8<br>36y (27.75-45.25)	EG: Pilates<br><br>CG: abdominal breathing + home exercises	No	Was high in EG (only 2 patients could not participate one session)	Balance (BBS); functional mobility (TUG); muscle strength of limbs (hand-held dynamometer); level of perceived balance confidence (ABC)	0, 8	• EG (postintervention): ↑ in all measures	No
Marandi et al. [47]<br>2013	To analyze the effects of Pilates exercises and aquatic training for a 12 week period on th dynamic balance of pwMS	(all women)<br><br>EG1=19;<br>EG2=19;<br>CG=19<br><br>(20-40y)	EG1: aquatic therapy;<br><br>EG2: Pilates;<br><br>CG: -	4 in each group	NA	Dynamic balance (SSST)	0,12	• EG1 and EG2 (postintervention): ↑ dynamic balance<br>• Both EG are better than CG: ↑ dynamic balance<br>• No difference between EG1 and EG2.	NA

↓: significant decrease; ↑: significant increase; ♀: woman; ♂: man; **10MWT**: 10-Meter Timed Walk Test; **1RM**: one-repetition maximum**; 2MWT**: 2-Minute Walk Test; **6MWT**: 6-Minute Walk Test; **7d-PAR**: Seven-day Physical Activity Recall Scale; **9HPT**: 9-Hole Peg Test; **ABC**: Activities Specific Balance Confidence Scale; **BBS**: Berg Balance Scale; **BDI**: Beck Depression Inventory; **BDNF**: brain-derived neurotrophic factor; **BICAMS**: Brief International Cognitive Assessment for MS; **BRB-N**: Brief Repeatable Battery of Neuropsychological Tests; **CNTF**: ciliary neurotrophic factor; **CE**: Closed eyes; **CG**: Control Group; **COPM**: Canadian Occupational Performance Measure; **DVD**: Digital Video Disc; **EDSS**: Expanded Disability Status Scale; **EG**: Experimental Group; **FES-I**: Falls Efficacy Scale International; **FIS**: Fatigue Impact Scale; **FRT**: Functional Reach Test; **FSS**: The Fatigue Severity Scale; **FSST**: Four Square Stept Test; **GDNF**: glial cell-derived neurotrophic factor; **GLTEQ**: Godin Leisure-Time Exercise Questionnaire; **HADS**: Hospital Anxiety and Depression Scale; **HADS-A:** Anxiety Subscale of the Hospital Anxiety and Depression Scale; **HADS-D**: Depression Subscale of the Hospital Anxiety and Depression Scale; **IL**: interleukin; **MFIS**: Modified Fatigue Impact Scale; **MS**: multiple sclerosis; **MSFC**: Multiple Sclerosis Functional Composite; **MSIS-29**: Multiple Sclerosis Impact Scale-29; **MSQoL-54**: Multiple Sclerosis Quality of Life-54 instrument; **MSWS-12**: Multiple Sclerosis Walking Scale; **MusiQoL**: Multiple Sclerosis International Quality of Life Questionnaire; **NA**: not available; **NRS**: numeric rating scale; **NT**: neurotrophin; **OE**: Open Eyes; **PASAT**: Paced Auditory Serial Addition Test; **PCI**: Physiological Cost Index; **PGIC**: Patients' Global Impression of Change Scale; **POMS-B**: Profile of Mood States – Brief Form; **PPMS**: primary progressive multiple sclerosis; **QoL**: Quality of Life; **QIDS**: Quick Inventory of Depressive Symptomatology; **pwMS**: persons with Multiple Sclerosis; **RRMS**: Relapsing-remitting multiple sclerosis; **RVGA**: Rivermead Visual Gait Assessment; **SLS**: Single Leg Stance; **SOCS**: suppressors of cytokine signalling; **SPMS**: secondary progressive multiple sclerosis; **SRT**: Sit-and-Reach Test; **SSST**: Six Spot Step Test; **STAI-Y1**: State Trait Anxiety Inventory; **STAI-Y2**: Trait Subscale of the State-Trait Anxiety Inventory; **T25FW**: Timed 25-Foot Walk; **TIS**: Trunk Impairment Scale; **TUG**: Timed Up and Go Test; **VAS**: Visual Analogue Scale; **y**: year.
